# Modifying the amino acids in conformational motion pathway of the α-amylase of *Geobacillus stearothermophilus* improved its activity and stability

**DOI:** 10.3389/fmicb.2023.1261245

**Published:** 2023-12-07

**Authors:** Yu-Ting Hu, Xi-Zhi Hong, Hui-Min Li, Jiang-Ke Yang, Wei Shen, Ya-Wei Wang, Yi-Han Liu

**Affiliations:** ^1^Pilot Base of Food Microbial Resources Utilization of Hubei Province, College of Life Science and Technology, Wuhan Polytechnic University, Wuhan, China; ^2^Key Laboratory of Industrial Fermentation Microbiology, Ministry of Education, Tianjin Key Laboratory of Industrial Microbiology, The College of Biotechnology, Tianjin University of Science and Technology, Tianjin, China

**Keywords:** amylase, conformational motion pathway, stability, kinetics, channel

## Abstract

Amino acids along the conformational motion pathway of the enzyme molecule correlated to its flexibility and rigidity. To enhance the enzyme activity and thermal stability, the motion pathway of *Geobacillus stearothermophilus* α-amylase has been identified and molecularly modified by using the neural relational inference model and deep learning tool. The significant differences in substrate specificity, enzymatic kinetics, optimal temperature, and thermal stability were observed among the mutants with modified amino acids along the pathway. Mutants especially the P44E demonstrated enhanced hydrolytic activity and catalytic efficiency (k_cat_/K_M_) than the wild-type enzyme to 95.0% and 93.8% respectively, with the optimum temperature increased to 90°C. This mutation from proline to glutamic acid has increased the number and the radius of the bottleneck of the channels, which might facilitate transporting large starch substrates into the enzyme. The mutation could also optimize the hydrogen bonding network of the catalytic center, and diminish the spatial hindering to the substrate entry and exit from the catalytic center.

## 1 Introduction

Alpha-amylase (E.C.3.2.1.1) is an enzyme that plays a crucial role in the hydrolysis of starch and glycogen by cleaving the alpha-1,4 glycosidic bond, resulting in the production of maltose and various oligosaccharides. Moreover, α-amylase has been widely applied in a variety of industrial processes, particularly in starch processing, food production, brewing, textile manufacturing, washing agents, biofuel production, and more. Due to the variety of industrial application environments for α-amylase, different requirements are placed on the enzyme properties, including optimal reaction temperature and thermal stability (Hussain et al., 2013; Gopinath et al., 2017; Farooq et al., 2021; Golgeri et al., 2022).

In the starch processing industry, enzymatic liquefaction of starch is commonly performed at elevated temperatures, which offers several advantages, including increased starch solubility and improved reaction rates, ultimately leading to reduced processing time (Oztug et al., 2020). However, the room temperature amylases exhibit poor thermal stability when exposed to high temperature conditions, resulting in a rapid decline in enzymatic activity (Gupta et al., 2003). *Geobacillus stearothermophilus* α-amylase, derived from a thermophilic spore-forming bacterium, belongs to a class of starch enzymes with significant potential for industrial applications (Srivastava and Baruah, 1986; Offen et al., 2015; Slavić et al., 2023). However, in modern starch processing processes, such as ethanol production, amylases are required to have higher temperature stability (the reaction temperatures typically exceeding 90°C). Currently, *G. stearothermophilus* α-amylase enzymes are still unable to meet the demands of high-temperature starch processing in terms of both enzymatic activity and thermal stability (Chen et al., 2018; Oztug et al., 2020). Therefore, it is imperative to further improve the activity of the enzymes and thermal stability.

Currently, some methods such as introducing Ca^2+^ salt or other ions into the amylases solution and introducing salt bridges into the enzyme molecules to improve their thermal stability were practiced (Nielsen and Borchert, 2000; Nielsen et al., 2003; Kumar et al., 2016; Ban et al., 2021). However, at the molecular level, the conformational flexibility and rigidity of enzyme molecules play pivotal roles in determining their stability (Offen et al., 2015; Zhu et al., 2023). The conformational motion pathway of an enzyme molecule describes its movement process, and the amino acids in the pathway might influence the patterns of movement (Alikhajeh et al., 2010). Neural Relational Inference (NRI) is a graph neural network model that can effectively identify the key residues and metastable pathways of enzyme molecules through molecular dynamics simulations (Zhu et al., 2022; Hong et al., 2023). These residues are closely related to the flexibility, rigidity, stability, and activity of the enzyme’s molecular structure.

In this study, we tentatively utilized the molecular dynamics simulations of α-amylase from *G. stearothermophilus* in an aqueous phase to predict the motion pathways within the loop regions by using the NRI model. Furthermore, leveraging deep learning techniques based on structural information, key amino acid residues along these conformational pathways will be engineered to investigate changes in enzymatic properties and substrate specificity, and also to obtain a novel α-amylase variant with high activity and thermal stability that meets industrial production requirements.

## 2 Materials and methods

### 2.1 Conformational motion pathways predicted by NRI

The molecular dynamics simulation of *G. stearothermophilus* α-amylase GsamyA (GenBank accession number AF032864.1) was conducted under the Amber99SB-ILDN force field. Initially, a reaction box was set up containing 38,651 TIP3P water molecules and 11 sodium ions. Subsequently, temperature equilibration (NVT) was conducted for 5 ns at 50°C, followed by pressure equilibration (NPT) for 1 ns. Finally, a 200 ns molecular dynamics simulation was performed using the NRI mathematical model, allowing for the analysis of residue dynamics. In the simulations mentioned above, the temperature coupling method is V-rescale, the pressure coupling method is Berendsen.

### 2.2 Modifications for amino acids chosen from motion pathway

The mutagenesis based on the aforementioned amino acids along the motion pathway was conducted, respectively. The protein structure of the enzyme was predicted using C-I-TASSER (Contact-guided Iterative Threading ASSEmbly Refinement), an enhanced version of I-TASSER that offers high-accuracy predictions of protein structure and function (Zheng et al., 2021).

Proceeding further, we employed a state-of-the-art, deep learning tool based on the three-dimensional structure of the protein predicted above. This tool, MutCompute, is freely available for academic use at www.mutcompute.com. This tool, a 3D convolutional neural network trained to establish connections between amino acids and their neighboring chemical microenvironments, guided the identification of innovative gain-of-function mutations in the docking model. Briefly, the tool rebuilt the neural network architecture and then, to further improve the accuracy, incorporated hydrogen atoms to explicitly recapitulate local hydrogen bonding networks, and added biophysical annotations such as partial charge and solvent accessibility to each atom (Shroff et al., 2020).

The amino acids in the conformational motion pathway of enzyme were predicted by NRI model as described above. The top-10 possible pathways were selected, and imputed into the MutCompute tool to get the candidate mutations. Through this approach, we identified amino acid mutations with the highest potential to enhance both the activity and stability of the amylase enzyme.

### 2.3 Construction and expression of amylase genes and mutants

The wild-type *G. stearothermophilus* α-amylase gene GsamyA was artificially synthesized based on the available sequence (GenBank accession No. AF032864.1) and subsequently cloned into the expression vector pET28a to generate the recombinant expression plasmid, pET28-amy. To introduce targeted mutations in the GsamyA amylase gene, two pairs of homology arm primers were designed utilizing the known coding sequence as a reference. The mutated sites of the amylase and the specific sequences for the inverse PCR primers were listed in Table 1. The point mutation was introduced into the gene by using inverse polymerase chain reaction (inverse PCR), with the recombinant plasmid pET28-amy as a template. The inverse PCR was conducted according to the description by Edelheit et al. (2009). Subsequently, a one-step cloning technique (One Step Cloning Kit, Vazyme Biotech, China) was conducted according to the description by the manufacture to form the recombinant plasmids carrying the mutant genes.

**Table 1:** The PCR primers used to construct the mutants.

Primers	Sequence (5′–3′)
P44E-F1	TACAAAGGATTGAGCCGCAGCGACGTAGGGTA
P44E-R1	GCTGCGGCTCAATCCTTTGTAAGCGGGCGGCA
L202I-F1	CGCAGCGACAATGGGTACGGAGTATACGACTT
L202I-R1	TCCGTACCCATTGTCGCTGCGGCTTGTTCCTT
K345I-F1	AAAGCTCAACTGCTTCAAGCCATTCAAGCCGC
K345I-R1	GGCTTGAAGCAGTTGAGCTTTTGTTCCATATT
T354C-F1	GACCATAAAGCTGGCGCTGACGGCACGGAATG
T354C-R1	GTCAGCGCCAGCTTTATGGTCGAACACGACAT
Q356P-F1	GACCTTGATTATGATCATCCCGAAGTCGTGAC
Q356P-R1	GGGATGATCATAATCAAGGTCGGCATACATTA
Y359T-F1	ACTGGCAAGGAACTATTTACCGTCGGGGAATA
Y359T-R1	GGTAAATAGTTCCTTGCCAGTCTGAGAACGCA
C361M-F1	AACAAGTTGGAGAATTACATTACGAAAACAGA
C361M-R1	AATGTAATTCTCCAACTTGTTGATGTCATAGC
F363H-F1	GGCCAATCTCTGGAATCATGGGTTGATCCATGGTT
F363H-R1	CCATGATTCCAGAGATTGGCCGGGTTCGGTGTCAT
Y364W-F1	ACGCTGGAGAAACTTTCTATGACCTTACCGGCAA
Y364W-R1	TCATAGAAAGTTTCTCCAGCGTGTTGTTTGCCAA
D366L-F1	ACTAAGGGTACTTCTCCTAAGGAAATTCCTTCGCTGAAAAGCAA
D366L-R1	TTCCTTAGGAGAAGTACCCTTAGTGCCATAATAGTCACCATAAA

The plasmid was transformed into *Escherichia coli* BL21(DE3) by chemical methods, and the colonies containing amylase gene were selected from LB medium plate (containing 50 μg/mL kanamycin). For inducible expression of amylase, a single colony was picked into 5 mL LB medium, incubated at 37°C, 180 rpm for 6–8 h. Then the liquid culture was inoculated in 500 mL LB liquid medium (containing 50 μg/mL kanamycin) at a ratio of 1:100. The bacteria were incubated at 37°C until the A_600_ value was 0.6. Then, 0.05 mmol/L IPTG was added to induce the expression of amylase gene, and the culture was induced at 16°C for 15 h. The bacteria were collected by centrifugation and resuspended in 20 mL lysis buffer (Tris-HCl, pH 7.5, 0.5 mmol/L MgSO_4_, 0.1 mmol/L MnCl_2_); After ultrasonic disruption of the bacterial cells, the supernatant containing the target amylase was collected by centrifugation and subsequently purified using a Ni-agarose column. The purified amylase was then analyzed by SDS-PAGE electrophoresis and its protein content was determined using the Bradford assay. The determination of amylase activity employed the 3,5-dinitrosalicylic acid (DNS) method to measure reducing sugar content. One unit (U) of amylase activity is defined as the amount of enzyme required to hydrolyze starch and produce 1 μmoL glucose per minute under specific assay conditions.

### 2.4 Determination of the activity and kinetic parameters of enzymes on different substrates

The hydrolytic activities of the wild-type and mutants towards different substrates, including soluble starch, amylopectin, pullulan, and potato starch, were measured. The reaction mixture containing 50 μL properly diluted enzyme and 500 μL substrates with 1.0% (w/v) concentration was incubated at 60°C for 5 min and then the amount of the reducing sugar liberated from the substrates was determined by the DNS method. The hydrolysis products of starch were analyzed by thin layer chromatography (TLC). In this process, the components were separated on silica gel plates with the developing solvent N-butyl alcohol, isopropyl alcohol, acetic acid and water with the ratio of Vn:Vi:Va:Vw = 7:5:2:4, and then visualized by phosphoric acid chromogenic agent. Enzyme kinetics measurements were performed using various concentrations (0.1–5.0 mg/mL) of soluble starch as substrate and the amylase activity after a 5-min reaction under optimal temperature. The kinetic parameters K_M_, V_max_, k_cat_, and k_cat_/K_M_ were obtained through data analysis using Lineweaver-Burk double reciprocal plots.

### 2.5 Determination of optimum temperature and temperature stability of enzymes

In order to ascertain the optimal temperature for enzymatic activity, the reaction mixtures described as above experiments were incubated at various temperatures ranging from 30°C to 90°C, and the enzyme activity was measured, respectively. To evaluate thermal stability, the enzyme solution was appropriately diluted and subjected to a 1-h treatment within the temperature range of 30°C to 90°C, and then the remaining enzyme activity was measured under the general conditions with 60°C temperature. To evaluate the half-life of mutants, the enzymes were incubated under 60°C, and the remaining activities were checked at intervals. Comparing to the highest activity, which was designated as 100% activity and illustrated as relative activity, the data were normalized. All of the values presented in graphs and tables are the means of three replicates.

### 2.6 Analysis of channel and starch interactions in the catalytic center of enzymes

Based on the available structural information, the protein channel of both the wild-type and mutants were examined. The analysis utilizes the CAVER software (Chovancova et al., 2012) for tunnel and channel analysis and visualization in protein structures. With the assistance of the PLIP website, the protein-ligand interaction profiler was detected and visualized (Adasme et al., 2021). The force maps of the interactions between adjacent sugar substrate and the corresponding residues surrounding the enzymatic cleavage site were showcased by using PyMOL.^1^

## 3 Results

### 3.1 Loop segments and conformational motion pathway of enzymes

The three-dimensional structure of *G. stearothermophilus* α-amylase revealed that the loop segments are primarily located between Domain A and B, as well as between Domain A and C (Table 2). A total of 23 loop segments were extracted from this amylase, with 17 segments in Domain A and 6 segments in Domain B. The catalytic residues D232, E262, and D329 involved in starch hydrolysis are all located within these loop regions and spatially positioned within an active pocket formed by Domain A and B. Within the vicinity of these catalytic residues, a total of 37 amino acids located within the loop structures contribute to the formation of the active pocket responsible for starch hydrolysis.

**Table 2:** Sequence characteristics of the loop segments in the amylase.

Loop segments	Structural domain	Catalytic pocket residues	Possible motion residues	Catalytic residues
1-AAPFNPT-7	A			
10-QYFEWYLPDDGT-21	A	W14		
36-LGINA-40	A			
43-LPPAYKGTSRSDVGYGVYDLYDLGEFNQKGTVRTKYGTK-81	A	T50,S51,53-SDV-55,Y57,G58,K71	P44	
94-AGMQ-97	A			
102-VVFDHKGGADGTE-114	B	105-DHKGGA-110	V103	
120-EVNPSDRNQEISGTY-134	B			
138-AWTKFDFPGRGNTYSSFKWRW-158	B	W139		
164-VDWDESRKLSRIY-176	B	164-VDW-166,		
178-FRGKAWDWEVDTEFGNYDYLMYA-200	B	E190,Y194,197-LMYA-200		
202-LDMDHP-207	B		L202,P207	
224-TNID-227	A			
231-LDAVKHIKFQ-240	A	D232,A233,K235,H236		D232
253-TGKPLF-258	A			
262-EYWSYDI-268	A	E262,264-WSY-266		E262
278-TDGTMS-283	A			
285-FDA-287	A			
299-SGGAFDM-305	A			
308-LMTNTL-313	A			
316-DQPHA-320	A			
323-TFVDNHDTEPGQALQSWVDPWFKPL-347	A	H328,D329,A335,L336	D341, 345-KPL-347	D329
351-FILTRQEGYPC-361	A		F351, I352, 354-TRQE-357,359-YPC-361	
363-FYGDYYGIPQYNIPSLK-379	A		F363, Y364, D366	

**Figure 1:**
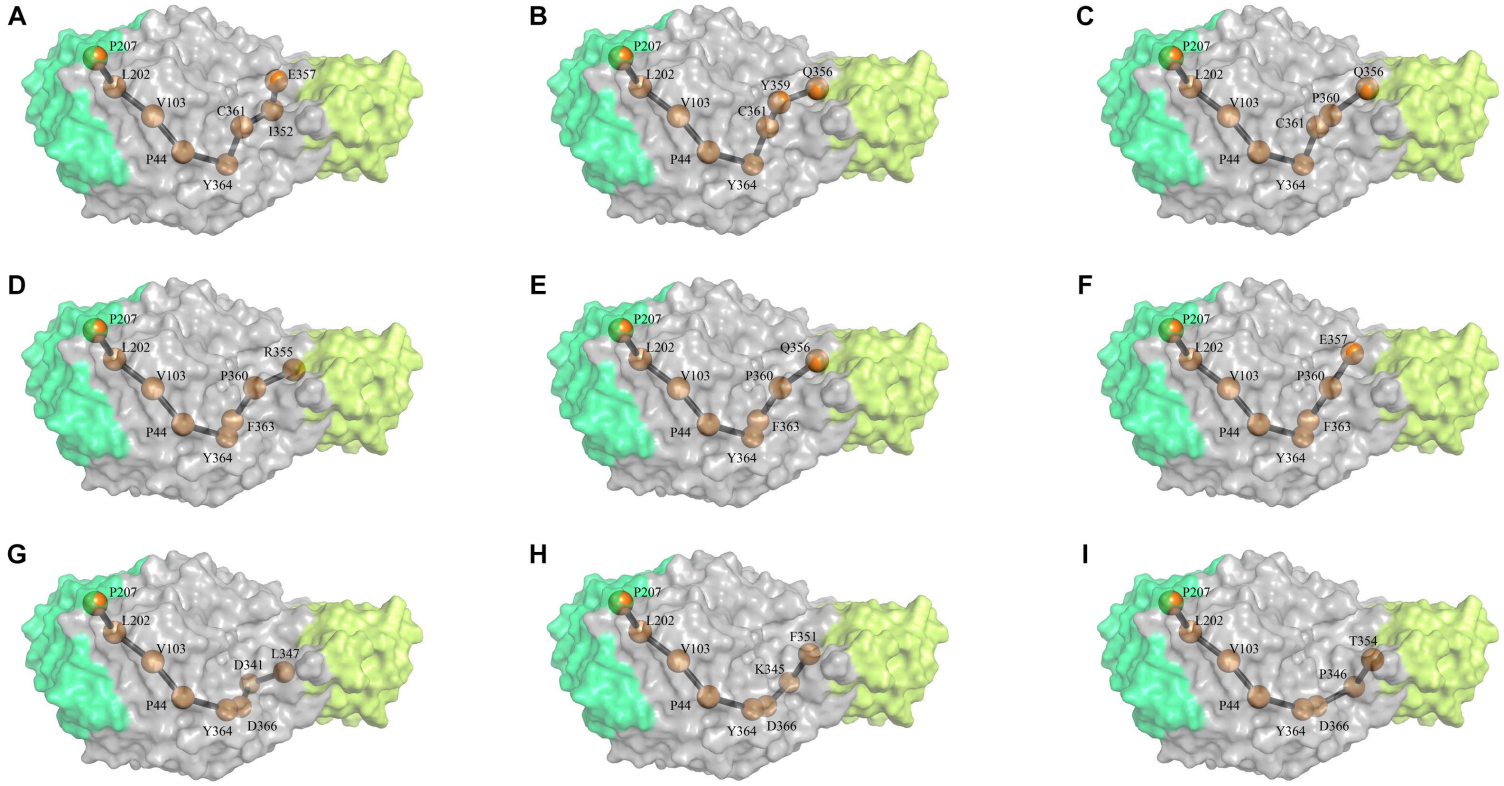
The most probable motion pathway of the amylase. Among the top 10 pathways calculated based on the NRI model, two pathways were found to be identical and merged. The **(A–I)** listed the top pathways as alphabet order. The position of the amino acid in structure similar to the 3-D structure of *G. stearothermophilus* amylase (RSCB PDB no. 6ag0).

Based on the NRI model, this study analyzed the motion pathways of the amylase and extracted the top 10 most probable routes (>80% possibility) for further analysis (Figure 1; Supplementary Table S1). Among these 10 highly probable pathways, the first five amino acid positions remain constant and are as follows: P207- > L202- > V103- > P44- > Y364. Variability in amino acid positions occurs at the 6th, 7th, and 8th positions along these trajectories. At position 6th, variations primarily occur between C361, F363, and D366. At position 7th, variations occur among D341, K345, P346, I352, Y359, and P360. At position 8th, variations take place between L347, F351, T354, P355, Q356, and E357 residues. It indicates that amino acids involved in conformational changes within the enzyme molecule are mainly located at positions P207- > L202- > V103- > P44- > Y364, and the P44 is occupies a “hinge” position during conformational changes (Figure 1).

### 3.2 Substrate preference and hydrolysis products by motion amino acid mutants of amylase

In order to improve the activity and stability of the enzyme molecule, the amino acids within the top 10 most probable motion pathways were analyzed by MutCompute,^2^ and the mutants were designed with maximum possibility to acquire improved activity and stability (Table 2). As shown by Table 3, the first five amino acids along the motion pathway remain relatively conserved, with mutations primarily occur between the 2nd (L202I) and 4th (P44E) position. However, there is greater variability observed in positions 6th, 7th, and 8th. Combined the candidate mutants in the 10 pathways, this study focused on ten specific mutants: P44E, L202I, K345I, T354C, Q356P, Y359T, C361M, F363H, Y364W, and D366L.

**Table 3:** Amino acids used for mutational modification on the movement pathways.

Pathways		The amino acids at each site							
A	SITES	207	202	103	44	364	361	359	356
	Original	P	L	V	P	Y	C	Y	Q
	The maximum possibility	P	I	V	E	W	M	T	P
B	SITES	207	202	103	44	364	361	360	356
	Original	P	L	V	P	Y	C	P	Q
	The maximum possibility	P	I	V	E	W	M	P	P
C	SITES	207	202	103	44	364	366	346	354
	Original	P	L	V	P	Y	D	P	T
	The maximum possibility	P	I	V	E	W	L	P	C
D	SITES	207	202	103	44	364	361	352	357
	Original	P	L	V	P	Y	C	I	E
	The maximum possibility	P	I	V	E	W	M	I	E
E	SITES	207	202	103	44	364	363	360	356
	Original	P	L	V	P	Y	F	P	Q
	The maximum possibility	P	I	V	E	W	H	P	P
F	SITES	207	202	103	44	364	363	360	355
	Original	P	L	V	P	Y	F	P	R
	The maximum possibility	P	I	V	E	W	H	P	R
G	SITES	207	202	103	44	364	363	360	357
	Original	P	L	V	P	Y	F	P	E
	The maximum possibility	P	I	V	E	W	H	P	E
H	SITES	207	202	103	44	364	366	345	351
	Original	P	L	V	P	Y	D	K	F
	The maximum possibility	P	I	V	E	W	L	I	V
I	SITES	207	202	103	44	364	366	341	347
	Original	P	L	V	P	Y	D	D	L
	The maximum possibility	P	I	V	E	W	L	D	M

**Figure 2:**
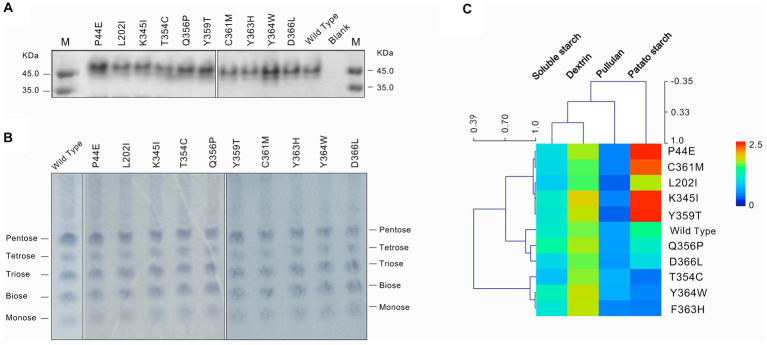
Expression and substrate characterization of the wild-type and mutants. **(A)** The protein of the wild-type and mutants of amylase checked by SDS-PAGE. **(B)** Thin layer chronographs of the products of starch hydrolyzed by amylases. **(C)** Activity of amylases on substrates of soluble starch, dextrin, pullulan and potato starch. The average linkage clustering and the Pearson correlation among the substrates and mutants were calculated.

The point mutation was introduced into the amylase gene by inverse PCR. After IPTG inducible expression of these mutant genes, were conBoth the wild-type and mutants achieved active expression (Figure 2A). The TLC analysis of starch hydrolysis products by both the wild-type and mutants revealed the presence of monosaccharides, disaccharides, and oligosaccharides with a degree of polymerization (DP) ranging from 1 to 5. Furthermore, no significant changes observed in the composition of products among different mutants (Figure 2B). The differences in enzymatic activities between the wild-type and mutants were evidenced using soluble starch, amylopectin, pullulan, and potato starch as substrates (Figure 2C; Table 4). Overall, the highest enzymatic activity was observed when amylopectin was utilized as the substrate, whereas the lowest activity was observed with pullulan. When potato starch was used as the substrate, mutants P44E, K345I, Y359T, and C361M exhibited significantly increased hydrolytic capacity compared to the wild-type by 95.0, 68.0, 74.0, and 33.0%, respectively. In contrast, mutants T354C, and F363H showed decreased hydrolytic capacity by 72.0% and 55.0%, respectively. These observations not only suggest possible structural heterogeneity among amylopectin, potato starch, soluble starch, and pullulan but also indicate distinct spatial differentiation among mutants particularly within the catalytic pocket.

**Table 4:** Enzymatic activities of the wild-type and mutants to different starch substrates.

	Soluble starch (×10^2^ U/μg)	Dextrin (×10^2^ U/μg)	Pullulan (×10^2^ U/μg)	Potato starch (×10^2^ U/μg)
Wild-type	1.14	1.64	0.62	1.44
L202I	0.86	1.56	0.33	1.78
Q356P	1.34	1.75	0.60	1.16
T354C	0.79	1.69	0.72	0.41
P44E	0.99	1.7	0.48	2.80
K345I	1.24	1.91	0.47	2.42
C361M	0.95	1.59	0.47	1.91
F363H	1.07	1.81	0.48	0.65
Y359T	1.08	1.82	0.33	2.50
D366L	0.95	1.62	0.58	0.97
Y364W	1.11	1.66	0.72	0.94

### 3.3 Optimal temperature and thermal stability of wild-type amylase and mutants

**Figure 3:**
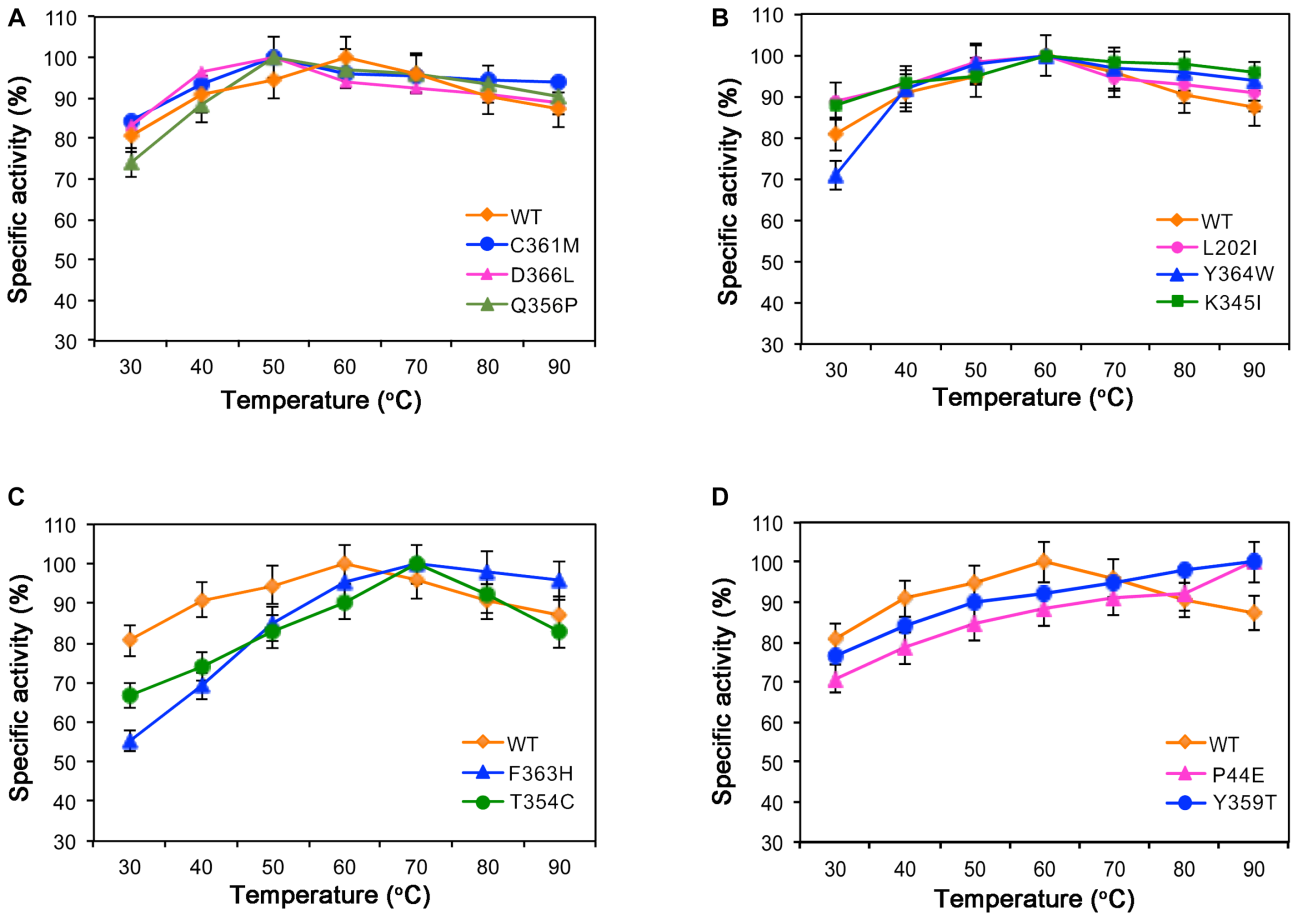
Enzymatic activities of the wild-type (WT) and mutants at different temperatures. **(A)** The optimal temperature for mutants C361M, D366L, and Q356P is 50°C. **(B)** Mutants L202I, Y364W, and K345I exhibit optimal activity at 60°C. **(C)** Mutants F363H and T354C have an optimal temperature of 70°C. **(D)** Mutants P44E and Y359T display their highest activity at 90°C.

The optimal temperature for the wild-type was found to be 60°C. Within the range of 50°C to 90°C, both the wild-type and mutants maintained over 80% of their maximum enzymatic activity, indicating that they are mid-temperature amylases. Mutants Q356P, C361M, and D366L exhibited a decrease in their optimal temperature to 50°C (Figure 3A). However, mutants L202I, K345I, and Y364W exhibited similar optimal temperature to the wild-type (Figure 3B). Conversely, mutants F354C and F363H demonstrated an increase in their optimal temperature to 70°C (Figure 3C). Mutants P44E and Y359T displayed a further elevation in their optimum temperature to 90°C (Figure 3D).

**Figure 4:**
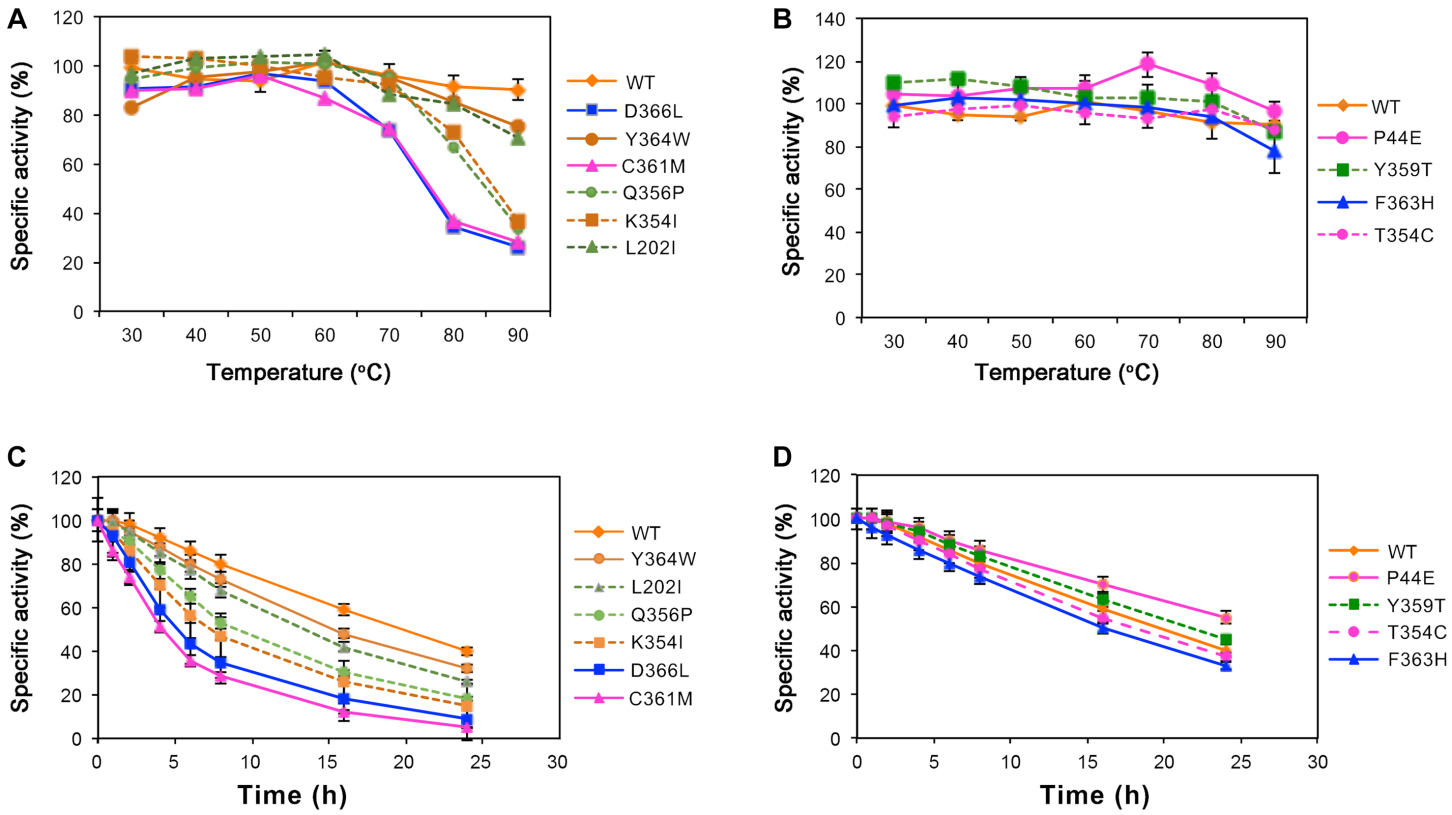
Stability of the wild-type (WT) and mutants at different temperatures. The residual enzymatic activity of amylase was measured after incubation for 1-h at various temperatures. **(A)** Mutants L202I, K354I, Q356P, C361M, Y364W, and D366L. **(B)** Mutants P44E, Y359T, F363H, and T354C. **(C, D)** The specific activity of the wild-type (WT) and mutants after incubated at 60°C.

The thermal stability of enzyme molecules was investigated at various temperatures, revealing distinct patterns between the wild-type and mutants (Figure 4). At lower temperatures, both the wild-type and mutants displayed exceptional stability. After a 1-h treatment at 60°C, their residual enzymatic activity remained above 80%. However, when the temperature exceeded 60°C, significant variations in enzyme stability were observed. Following a 1-h treatment at 80°C, mutants C361M and D366L exhibited a decrease in activity by 36%. Subsequently, after treatment at 90°C for 1-h, the remaining enzymatic activity for mutants K354I, Q356P, C361M, and D366L dropped below 40% (Figure 4A), and the half-life for them were 7.5-, 8.0-, 4.0-, and 5.0-h, respectively, when incubated under 60°C which were significantly lower than the 18.2-h of the wild-type (Figure 4C). However, mutants P44EY, Y359T, and T354C demonstrated improved temperature stability. After 1-h of treatment at 90°C, mutant P44E maintained more than 90% of the enzyme activity, and the half-life was 25.0-h, which was significantly better than the wild-type (Figures 4B,D).

### 3.4 Kinetic parameters of wild-type amylase and mutants

The kinetic parameters of the wild-type and mutants are shown in Table 5. Mutants P44E, Y359T, and D366L exhibited significantly increased substrates affinity. The K_M_ values decreased by 39.1, 54.3, and 33.7% compared to the wild-type, respectively. Mutants L202I, K345I, and F363H exhibited a decrease in substrate affinity, as indicated by an increase in K_M_ values of 14.1, 17.4, and 26.1%, respectively. Furthermore, mutants P44E, K345I, and Y359T exhibited an enhancement in catalytic efficiency (k_cat_/K_M_), with increases of 93.8%, 5.0%, and 27.2% respectively, compared to the wild-type.

**Table 5:** Kinetic parameters of the wild-type and mutants.

Enzyme	V_max_ (μmol/min)	K_M_ (mg/mL)	k_cat_ (1/s)	k_cat_/K_M_ (mL/mg/s)
Wild type	1.19	0.92	539.14	585.48
D366L	0.72	0.61	345.65	565.50
K345I	1.28	1.08	659.83	610.95
T354C	0.76	0.96	316.46	329.64
Y359T	1.26	0.42	312.78.	744.71
Q356P	0.96	0.86	375.26	436.35
L202I	1.12	1.05	576.23	548.79
Y364W	1.02	0.91	463.67	509.53
C361M	0.87	0.91	507.62	557.82
F363H	0.90	1.16	456.25	393.32
P44E	1.32	0.56	635.35	1134.55

### 3.5 Protein channels and interactions between substrates and catalytic center

**Figure 5:**
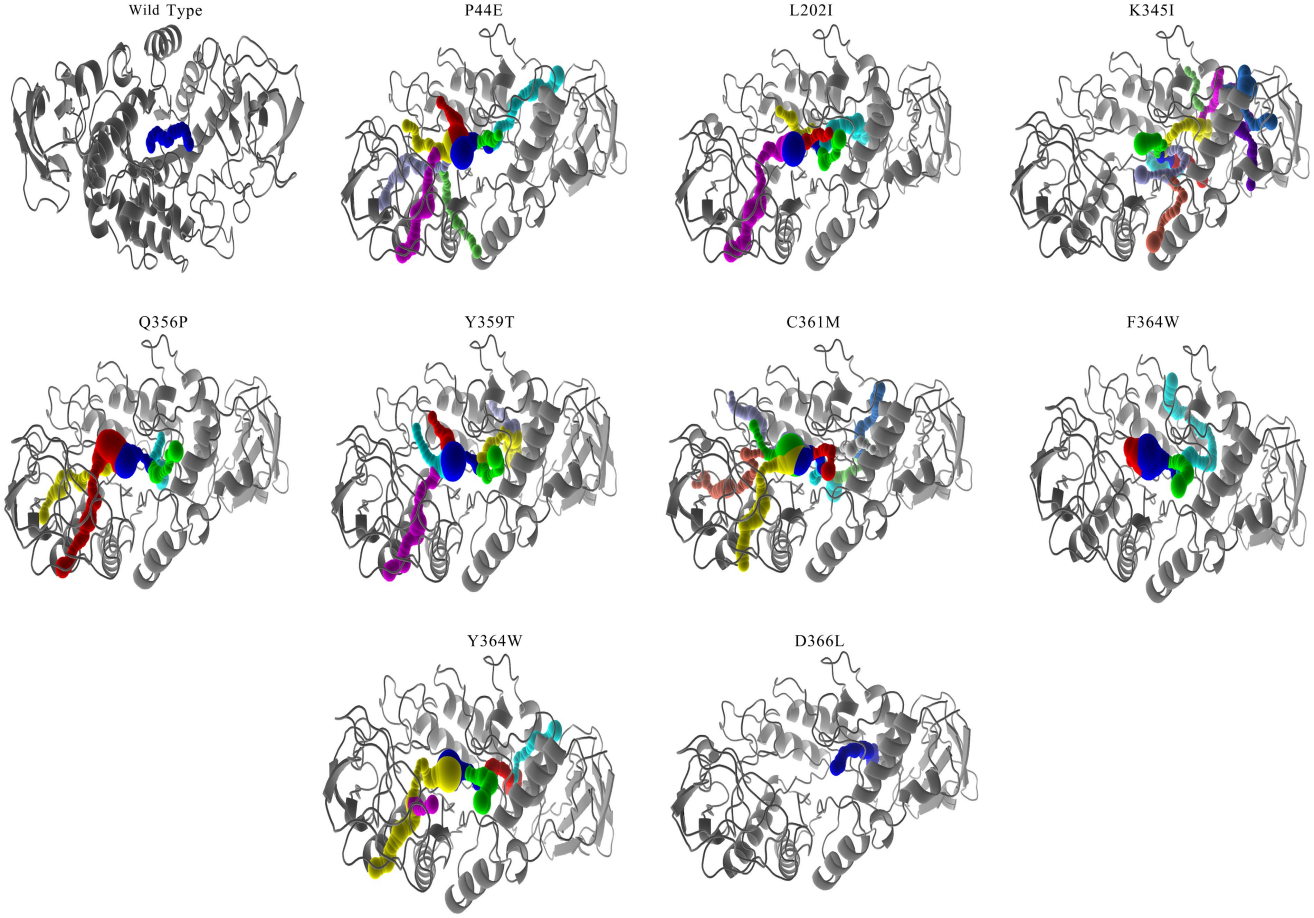
Channel analysis of the wild-type and mutants. The molecular pathways within the amylase molecule were calculated using CAVER software (Chovancova et al., 2012). Mutant T354C did not showcased due to its exhibit no channel.

Protein tunnels and channels play a crucial role in the flexibility, stability, and enzymatic activity of proteins. A comprehensive analysis of the protein channels in both the wild-type and mutants was conducted (Table 6; Figure 5; and Supplementary Figure S1). As illustrated in the figures, significant differences were observed between the wild-type and mutants with respect to channel quantity, radius, and length. The wild-type enzyme contains only one channel, while most of mutants displayed an increase in the number of channel (Figure 5). For instance, mutants P44E, K345I, and Y359T had 8, 5, and 7 channels, respectively. The highest number of channels was found in mutant C361M with up to ten distinct channels. However, mutant T354C appeared to lack any observable channels (Table 5). The lengths and radii of the channels were crucial factors influencing substrate and ion transport. Distinct differences in the length and the radii were evident both among different enzyme molecules and within individual enzymes across various channel regions. The wild-type has a channel length of 16.34 Å, while the longest channel was observed in mutant Y364W, measuring 45.02 Å. In contrast, mutant Q356P displayed shorter channels with a length of only 14.50 Å. Mutant P44E possessed the largest bottleneck radius at 1.00 Å, whereas mutant D366L had a significantly smaller channel bottleneck radius of only 0.71 Å (Table 5; Figure 5).

**Table 6:** Characteristics of the protein channel of the wild-type and mutants.

	Channel number	Length (Å)	Bottleneck radius (Å)	Bottleneck residues
WT	1	16.34	0.93	W264/N327/E262/D329/L336/R230/F324/F285
P44E	8	29.97	1.00	Y349/F363/V325/D326/F324/V362/C361/D366/K345/N327
L202I	6	33.00	0.84	P360/L353/V362/T7/C361/L386/R389/M8/I38/I352
K345I	5	16.18	0.82	H328/W264/E262/N327/R230/W42/D329/F285
T354C	0	–	–	–
Q356P	5	14.50	0.81	A287/F324/W264/Y266/F285/H290/N327/D286
Y359T	7	34.00	0.76	L386/T7/L353/R389/I38/V362/G37/P360/P6
C361M	10	40.42	0.78	W264/H328/E262/N327/R230/F285/D329/W42
F363H	4	23.83	0.78	W264/H328/E262/N327/R230/D329/Y57/W42/Q10
Y364W	6	45.02	0.73	H328/N327/W264/E262/W42/R230/D329/Q10/F285/F324
D366L	1	32.27	0.71	T7/L386/L353/R389/I38/V362/P360/G37

**Figure 6:**
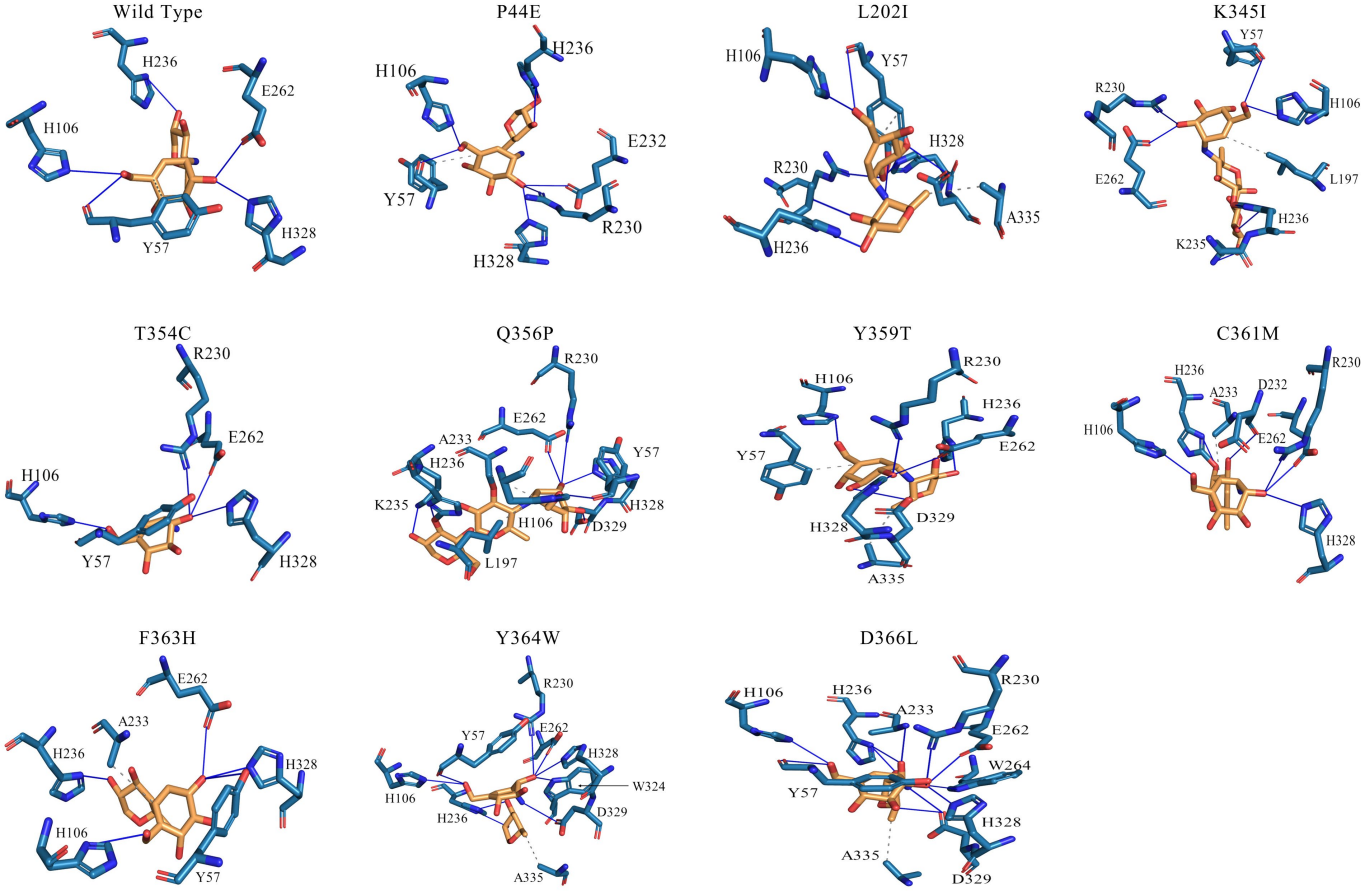
Interactions between the amino acids in the active site of the wild-type and mutants. The two adjacent sugar substrate binding sites, with corresponding residues surrounding the cleavage site were showcased. In this diagram, solid blue lines represent hydrogen bond interactions, while gray dashed lines represent hydrophobic interactions.

The interactions between substrates and amino acids within the active pockets of both the wild-type and mutants were analyzed, and the specific interactions occurring between the amino acids located before and after the cleavage site on the substrate molecule were illustrated (Figure 6; Supplementary Figure S2). In the wild-type enzyme, H236 forms an essential hydrogen bond with the −3 subsite, while E262, H328, and H106 each establish distinct hydrogen bonds with the −2 subsite. Y57, an aromatic amino acid, robustly interacted with the sugar ring at the −1 subsite to further enhance the interaction between substrate and enzymes. Mutants T354C, Q356P, and F363H introduce cysteine (C), proline (P), or histidine (H) as replacements for existing amino acids. It leads to a much more compacted enzyme structure overall due to the introduction of hydrophilic, basic amino acids or subamino groups (P) along the pathway, and formed a more complex hydrogen bonding network between amino acids in the catalytic center and substrate molecules (Figure 6). Thus might decrease the enzyme-substrate conversion rate, results and reduced enzymatic activity. Mutants P44E, K345I, and Y359T exhibit significant changes in the hydrogen bonding network within the catalytic center due to the substitution of more hydrophobic amino acids. These substitutions introduce both hydrogen bonds and hydrophobic interactions. Amino acid Y57 was becoming distant to the sugar ring (4 Å), and eliminated the aromatic stacking interactions with the sugar rings (Figure 6; Supplementary Figure S2).

## 4 Discussion

Typically, *G. stearothermophilus* α-amylase consists of three structural domains, Domain A, Domain B, and Domain C (Kikani and Singh, 2022). Domain A is primarily characterized by a highly symmetrical arrangement of α-helices and β-sheets forming the classical (β/α)_8_ TIM barrel structure (Suvd et al., 2001; Offen et al., 2015). Domain B, located between the third β-sheet and the third α-helix of the TIM barrel, has an irregular β-sheet structure. The region connecting Domains A and B forms a flexible substrate-binding cleft that accommodates the catalytic activity pocket. In our study, it is further evident that the loop regions located between Domain A and B of *G. stearothermophilus* α-amylase collectively form the catalytic pocket. The catalytic residues D232, E262, and D329 are situated at the bottom of this pocket (Table 1). Domain C encompasses the C-terminal region of the amylase and contributes to binding with larger molecular substrates (Mehta and Satyanarayana, 2015; Kikani and Singh, 2022).

The structural arrangement of the enzyme contains calcium binding sites that facilitate interactions between Ca^2+^ and the surrounding amino acids. These interactions are essential for maintaining the thermal stability of the enzyme molecule. During the initial stages of production, it was a common practice to introduce a specific quantity of Ca^2+^ salt or other metal ion into the amylase solution as a means to enhance its thermal stability (Nielsen and Borchert, 2000; Nielsen et al., 2003; Kumar et al., 2016). Subsequently, there has been a growing interest in enhancing the thermal stability of the enzyme molecule by introducing salt bridges into the amylase structure (Ban et al., 2021).

During the study of *Bacillus amyloliquefaciens* α-amylase, Domain B was found to be important for the overall flexibility of the enzymes, suggesting that Domain B rigidity provides a significant improvement in thermostability (Alikhajeh et al., 2010). The thermal stability of amylase can be significantly enhanced through structural-based rational design approaches. These strategies involve reducing the free energy associated with enzyme molecule folding, as well as incorporating targeted amino acid substitutions and mutations (Ben Ali et al., 2006; Deng et al., 2014; Yuan et al., 2023). During the dynamic motion of enzyme molecules, conformational motion pathways play a pivotal role in determining the patterns and rate of protein motion. These pathways involve structural changes that occur within the molecule (Hong et al., 2023). In this study, we tentatively modified the amino acids in the motion pathway of *G. stearothermophilus* α-amylase and investigated their effect on the activity and thermal stability of enzyme.

In order to correctly predict the motion pathway, the NRI, a graph neural network model MutCompute that holds potential for application in molecular dynamics was used. Although previous research reported that the strategy such as conformational ensembles could enhance our understanding of biomolecular motion, recognition, and allostery (Fenwick et al., 2011), the NRL model can effectively identify the residues accountable for protein motion within both aqueous and gaseous environments (Zhu et al., 2022). As shown by Figure 1 and Supplementary Table S1, the optimal movement pathways predicted by the NRI model have high possibility (>80%). Among the top 10 most probable pathways, the amino acids in the first five positions of each pathway were conserved, specifically P207- > L202- > V103- > P44- > Y364. This reflects that the motion pathway generated by the NRI model is reliable, and also the identification of these specific residues holds significant implications for understanding the flexibility, rigidity, as well as the stability of enzymes.

The C-I-TASSER software, a deep learning tool based on protein 3D protein structure, was utilized to predict the enzyme’s structure and guide the identification of novel gain-of-function mutations that were predicted to have the highest probability of improving both activity and stability (Shroff et al., 2020; Zheng et al., 2021). As shown by the results, mutants along the identified motion pathways exhibit significant differences in substrate specificity, enzymatic kinetics, optimal temperature, and thermal stability (Figures 2–4). Successfully, mutants P44E, K345I, and Y359T demonstrated enhanced hydrolytic activity and catalytic efficiency (K_cat_/K_M_) towards starch substrates compared to the wild-type (Figure 2). Especially on mutant P44E, which displayed enhancements in both optimal reaction temperature and thermal stability (Figures 3, 4).

Protein tunnels and channels are a series of cavities within the protein structure that play pivotal roles in facilitating protein enzyme activity and stability (Gouaux and Mackinnon, 2005; Damborský et al., 2007). This study systematically analyzed the protein tunnels and channels of both the wild-type and mutants by utilizing CAVER software (Chovancova et al., 2012), a robust tool renowned for its capabilities in the comprehensive examination and visualization of tunnels and channels within protein structures (Table 5; Figure 5; and Supplementary Figure S1). Significant differences were observed between the wild-type and mutants in terms of the channel quantity, radius, and length. However, the number of channels was not directly correlated with enzyme activity. The mutants with high numbers of channels do not means of the highest activity (Table 5). However, the radius at the bottleneck of channel seems positively related to the activity levels. Mutant P44E having highest activity on starch demonstrated the largest bottleneck radius at 1.00 Å. Wider channels may be more adept at accommodating larger starch substrates and consequently exhibiting higher enzymatic activity.

**Figure 7:**
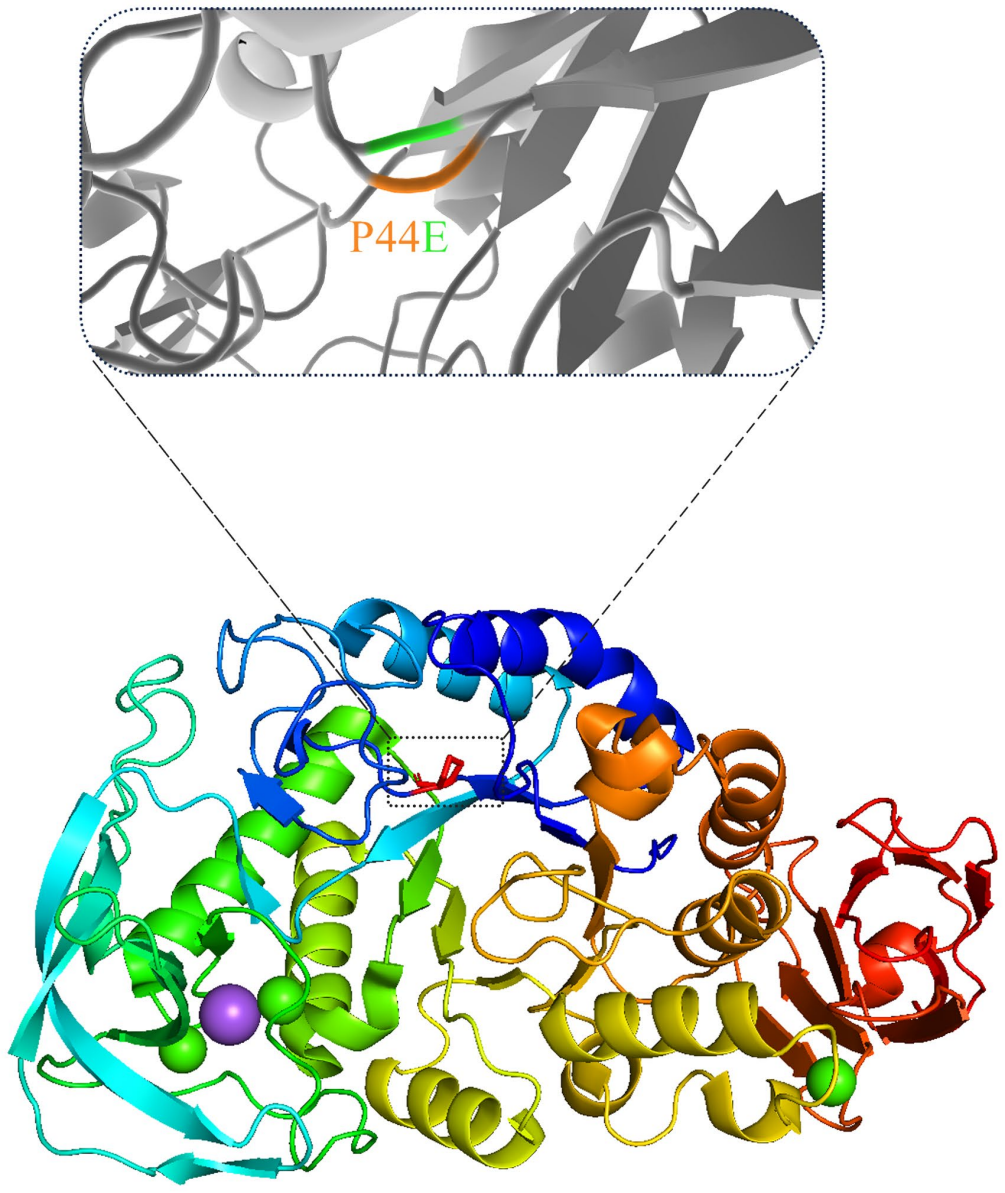
The spatial changes before and after the mutation. The position of the mutated amino acid proline (P) in the three-dimensional structure of mutant P44E was marked with dashed box.

The hydrogen bond network formed between substrate molecules and amino acids within the catalytic center of the enzymes play a vital role in influencing their activity and stability (Sebastiani et al., 2023; Yuan et al., 2023). Hydrophilic amino acids could increase the hydrogen bonding with substrates and vice versa. However, it is crucial to maintain a balance in the presence of hydrogen bonds as excessive bonding can impede substrate dissociation, leading to reduced enzyme activity. Consequently, the catalytic center of enzymes often exhibit an optimal composition of both hydrophilic and hydrophobic amino acids (Nielsen and Borchert, 2000; Ranjani et al., 2014). As shown by Figure 6, the amino acids within the active center of mutants Q356P and F363H form intricate hydrgen bonding networks with substrate molecules. This intricate network may lead to a substantial reduction in the conversion rate of substrate molecules within the active center, resulting in a notable decrease in enzymatic activity compared to the wild-type (Table 3). However, mutants P44E, K345I, and Y359T exhibit enhanced activity relative to the wild-type by substituting hydrophobic amino acids for their original hydrophilic and aromatic counterparts, which could bring a better balance on the hydrophilic and hydrophobic amino acids (Figure 6; Supplementary Figure S2). Especial for mutant P44E, the proline (P44) in the wild-type enzyme, positioned within the central region of the motion pathway, plays a crucial role as an intermediate “hinge” during conformational movements. Upon mutation of proline to glutamic acid (P44E), a hydrophilic amino acid substitution for the original hydrophobic residue occurs. This substitution leads to an enhancement in the conformational rigidity of the enzyme, consequently increasing its stability (Figure 6). It is worth noting that proline (P44) is positioned at the outer edge of the active pocket within the spatial structure (Figure 7). The subamino ring of the proline side chain contributes to the compression of the enzyme molecule’s conformation, resulting in hindrance to substrate entry and a decrease in enzymatic conversion rate. The mutation (P44E) has successfully alleviated this conformational hindrance while optimizing the hydrogen bonding network within the catalytic center.

The conformational flexibility and rigidity of the enzyme molecule play a decisive role in determining its overall stability (Dušan and Shina Caroline Lynn, 2018; Maria-Solano et al., 2018; Zhu et al., 2023). The generally used method was to substitute and mutate the amino acids in the surface of the protein to enhance the stability of amylase (Ben Ali et al., 2006; Deng et al., 2014; Yuan et al., 2023). Conformational motion pathways are the profile of the dynamic motion of enzyme molecules might act as switch plays a pivotal role in regulation of many essential biological pathways (Oyen et al., 2015; Hong et al., 2023). In this study, we tentatively modified the amino acids in the motion pathway of *G. stearothermophilus* α-amylase and investigated their effect on the activity and thermal stability of enzyme. Although the mutagenesis of residues located at flexible regions of kynureninase resulted even higher rate of the chemical reaction (Karamitros et al., 2022). That amino acid substitution allosterically affect the flexibility of the binding pocket, thereby impacting the rate of chemistry. Method used in this study has showed higher efficiency on targeting the key amino acids than the mutation on surface amino acids, and show dual roles on enhancing the enzymes stability and activity.

## 5 Conclusion

This study demonstrates the identifying and modifying amino acids in enzyme motion pathways using computational analysis with NRI and deep learning tools to obtain amylase mutants with improved activity and stability is a feasible strategy. The amino acid situated within the central region of the conformational motion pathway function as pivotal “hinge” positions that not only contribute to maintaining structural stability but also play a crucial role in facilitating substrate entry and exit from the catalytic center. It could be speculated that through rational design of these key amino acids, enzymes exhibiting substantial enhancements in both activity and stability might be obtained in the future works. These findings address the industrial demand for highly active and heat-resistant enzymes.

## Data availability statement

The original contributions presented in the study are included in the article/Supplementary material, further inquiries can be directed to the corresponding authors.

## Author contributions

Y-TH: Writing – original draft, Investigation, Methodology, Validation, Writing – review & editing. X-ZH: Formal analysis, Visualization, Writing – review & editing. H-ML: Methodology, Writing – review & editing. J-KY: Investigation, Methodology, Writing – review & editing. WS: Methodology, Writing – review & editing. Y-WW: Methodology, Writing – review & editing. Y-HL: Investigation, Writing – review & editing.

## Footnotes

^1^ https://pymol.org/2/

^2^ www.mutcompute.com
