# Supplementary material for: Modifying the amino acids in conformational motion pathway of the α-amylase of Geobacillus stearothermophilus improved its activity and stability
Source: Front Microbiol. 2023 Dec 7;14:1261245. doi: 10.3389/fmicb.2023.1261245 (PMC10740195; doi:10.3389/fmicb.2023.1261245)
Supplement: Supplementary file 1 [file Data_Sheet_1.docx]

**Supplementary Data**

**Table S1**. The most probable conformational motion pathways in amylase

| Items | Exercise paths and participating amino acids | Possibility  (%) |
| --- | --- | --- |
| A | P207->L202->V103->P44->Y364->C361->Y359->Q356 | 87.35 |
| B | P207->L202->V103->P44->Y364->C361->P360->Q356 | 86.03 |
| C | P207->L202->V103->P44->Y364->D366->P346->T354 | 84.76 |
| D | P207->L202->V103->P44->Y364->C361->I352->E357 | 83.87 |
| E | P207->L202->V103->P44->Y364->F363->P360->Q356 | 83.29 |
| F | P207->L202->V103->P44->Y364->F363->P360->R355 | 83.03 |
| G | P207->L202->V103->P44->Y364->F363->P360->E357 | 82.91 |
| H | P207->L202->V103->P44->Y364->D366->K345->F351 | 82.53 |
| I | P207->L202->V103->P44->Y364->D366->D341->L347 | 82.36 |


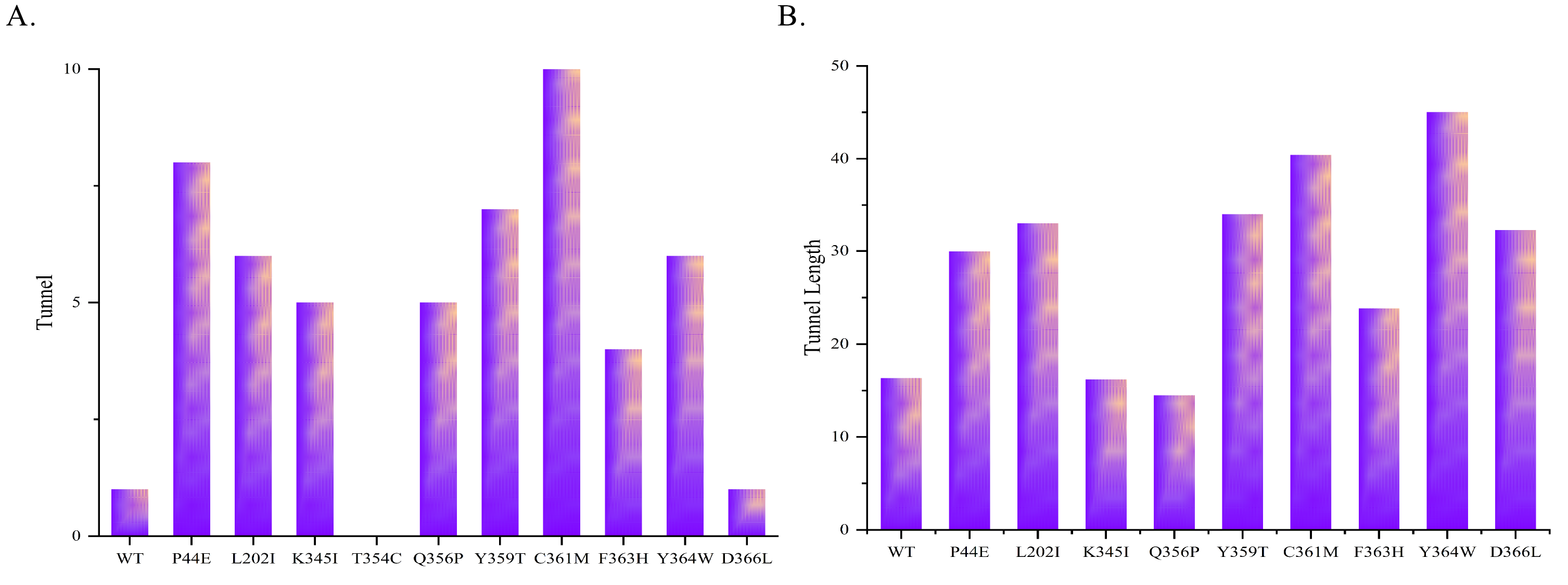


**Figure S1**. The number (**A**) and length of channels in the molecular structures of the wild-type and mutants. Since mutants T354C does not have any channels, it is not shown in **(B)**.


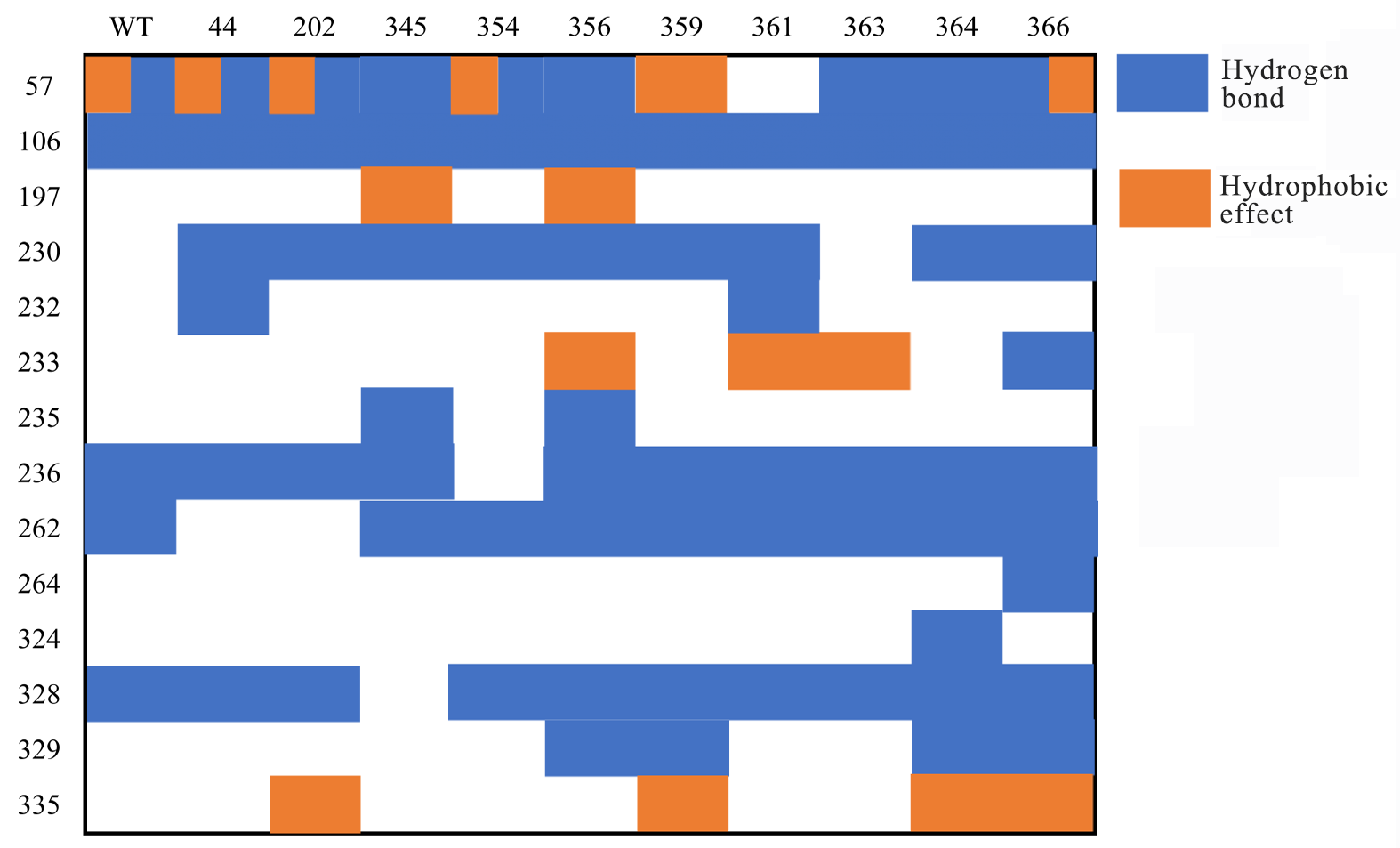


**Figure S2**. The types of forces provided by the amino acids in the active site when interacting with substrate molecules in different mutants. The x-axis represents different mutants, and the y-axis represents residue positions involved in interactions. In this diagram, orange blocks represent hydrophobic interactions, while blue blocks represent hydrogen bond interactions.
